# Quality control of cardiac magnetic resonance imaging segmentation, feature tracking, aortic flow, and native T1 analysis using automated batch processing in the UK Biobank study

**DOI:** 10.1093/ehjimp/qyae094

**Published:** 2024-09-16

**Authors:** Sucharitha Chadalavada, Elisa Rauseo, Ahmed Salih, Hafiz Naderi, Mohammed Khanji, Jose D Vargas, Aaron M Lee, Alborz Amir-Kalili, Lisette Lockhart, Ben Graham, Mihaela Chirvasa, Kenneth Fung, Jose Paiva, Mihir M Sanghvi, Gregory G Slabaugh, Magnus T Jensen, Nay Aung, Steffen E Petersen

**Affiliations:** William Harvey Research Institute, NIHR Barts Biomedical Research Centre, Queen Mary University of London, Charterhouse Square, London, UK; Barts Heart Centre, St Bartholomew’s Hospital, Barts Health NHS Trust, West Smithfield, London, UK; William Harvey Research Institute, NIHR Barts Biomedical Research Centre, Queen Mary University of London, Charterhouse Square, London, UK; Barts Heart Centre, St Bartholomew’s Hospital, Barts Health NHS Trust, West Smithfield, London, UK; Digital Environment Research Institute, Queen Mary University of London, London, UK; William Harvey Research Institute, NIHR Barts Biomedical Research Centre, Queen Mary University of London, Charterhouse Square, London, UK; Department of Computer Science, University of Zakho, Zakho, Kurdistan of Iraq, Iraq; William Harvey Research Institute, NIHR Barts Biomedical Research Centre, Queen Mary University of London, Charterhouse Square, London, UK; Barts Heart Centre, St Bartholomew’s Hospital, Barts Health NHS Trust, West Smithfield, London, UK; William Harvey Research Institute, NIHR Barts Biomedical Research Centre, Queen Mary University of London, Charterhouse Square, London, UK; Barts Heart Centre, St Bartholomew’s Hospital, Barts Health NHS Trust, West Smithfield, London, UK; Department of Cardiology, US Department of Veterans Affair Medical Center, Washington, D.C., USA; William Harvey Research Institute, NIHR Barts Biomedical Research Centre, Queen Mary University of London, Charterhouse Square, London, UK; Circle Cardiovascular Imaging Inc., Calgary, Canada; Circle Cardiovascular Imaging Inc., Calgary, Canada; Circle Cardiovascular Imaging Inc., Calgary, Canada; Circle Cardiovascular Imaging Inc., Calgary, Canada; William Harvey Research Institute, NIHR Barts Biomedical Research Centre, Queen Mary University of London, Charterhouse Square, London, UK; Barts Heart Centre, St Bartholomew’s Hospital, Barts Health NHS Trust, West Smithfield, London, UK; William Harvey Research Institute, NIHR Barts Biomedical Research Centre, Queen Mary University of London, Charterhouse Square, London, UK; William Harvey Research Institute, NIHR Barts Biomedical Research Centre, Queen Mary University of London, Charterhouse Square, London, UK; Barts Heart Centre, St Bartholomew’s Hospital, Barts Health NHS Trust, West Smithfield, London, UK; Digital Environment Research Institute, Queen Mary University of London, London, UK; School of Electronic Eng. & Computer Science, Queen Mary University of London, UK; Alan Turing Institute, The British Library, John Dodson House, London, UK; William Harvey Research Institute, NIHR Barts Biomedical Research Centre, Queen Mary University of London, Charterhouse Square, London, UK; Steno Diabetes Center Copenhagen, Cardiometabolic Disease Department, Borgmester Ib Juuls Vej 83, 2730 Herlev, Denmark; William Harvey Research Institute, NIHR Barts Biomedical Research Centre, Queen Mary University of London, Charterhouse Square, London, UK; Barts Heart Centre, St Bartholomew’s Hospital, Barts Health NHS Trust, West Smithfield, London, UK; Digital Environment Research Institute, Queen Mary University of London, London, UK; William Harvey Research Institute, NIHR Barts Biomedical Research Centre, Queen Mary University of London, Charterhouse Square, London, UK; Barts Heart Centre, St Bartholomew’s Hospital, Barts Health NHS Trust, West Smithfield, London, UK; Circle Cardiovascular Imaging Inc., Calgary, Canada; Alan Turing Institute, The British Library, John Dodson House, London, UK; Health Data Research UK, London, UK; National Institute for Health and Care Research, UK

**Keywords:** cardiac magnetic resonance imaging, automated image analysis, quality control, Shiny app, machine learning

## Abstract

**Aims:**

Automated algorithms are regularly used to analyse cardiac magnetic resonance (CMR) images. Validating data output reliability from this method is crucial for enabling widespread adoption. We outline a visual quality control (VQC) process for image analysis using automated batch processing. We assess the performance of automated analysis and the reliability of replacing visual checks with statistical outlier (SO) removal approach in UK Biobank CMR scans.

**Methods and results:**

We included 1987 CMR scans from the UK Biobank COVID-19 imaging study. We used batch processing software (Circle Cardiovascular Imaging Inc.—CVI42) to automatically extract chamber volumetric data, strain, native T1, and aortic flow data. The automated analysis outputs (∼62 000 videos and 2000 images) were visually checked by six experienced clinicians using a standardized approach and a custom-built R Shiny app. Inter-observer variability was assessed. Data from scans passing VQC were compared with a SO removal QC method in a subset of healthy individuals (*n* = 1069). Automated segmentation was highly rated, with over 95% of scans passing VQC. Overall inter-observer agreement was very good (Gwet’s AC2 0.91; 95% confidence interval 0.84, 0.94). No difference in overall data derived from VQC or SO removal in healthy individuals was observed.

**Conclusion:**

Automated image analysis using CVI42 prototypes for UK Biobank CMR scans demonstrated high quality. Larger UK Biobank data sets analysed using these automated algorithms do not require in-depth VQC. SO removal is sufficient as a QC measure, with operator discretion for visual checks based on population or research objectives.

## Graphical Abstract

**Graphical Abstract:**
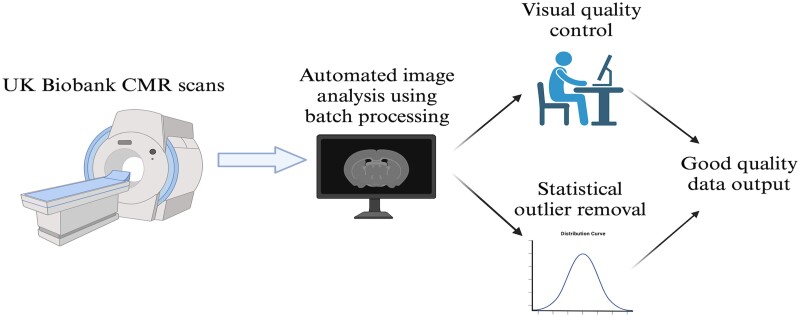
Created using Biorendr.com.

## Introduction

Cardiac magnetic resonance (CMR) imaging is considered the reference standard for assessing cardiac structure and function.^1,2^ Manual image segmentation is essential in deriving several quantitative measurements and is time-consuming and prone to error.^3^ Automated segmentation based on machine learning (ML) algorithms can improve the clinical workflow and increase the reproducibility of measurements by reducing intra- and inter-observer variability.^4,5^ However, quality assessment of the outputs generated by ML algorithms remains an essential step before they are deployed in clinical practice.

The quality of automated segmentation is generally assessed against a ground-truth reference, typically represented by manual expert annotations not always available in large data sets. Furthermore, the quantitative metrics used to evaluate the segmentation algorithms do not necessarily correlate with clinical acceptability.^6,7^ A quality assessment involving clinicians is thus essential to ensure the reliability and trustworthiness of ML outputs in clinical workflows. However, visual quality control (VQC) is a tedious, time-consuming, and often subjective process that relies on clinicians inspecting images and assigning scores. Furthermore, it requires multiple well-trained people to perform VQC, which is not always practical, especially for large data sets like the UK Biobank. Alternative automated QC techniques that do not require large, fully annotated data sets have been proposed in the literature, but these approaches do not guarantee the generalizability of the algorithm in unseen data.^8,9^

As current automated methods have become more accurate and successfully used for assessing several heart diseases,^10–12^ we hypothesized that it may be possible to visually QC only a subset of output data to assess the clinical acceptability and reliability of the ML algorithm. Here, we describe a QC process applied to a subset of UK Biobank participants enrolled in the COVID-19 study in which CMR images were analysed using automated batch processing algorithms. First, a team of six clinicians with extensive experience in interpreting and analysing CMR images visually assessed the automated outputs by assigning a quality score. Then, the automated measurements of cardiac function and structure related to the images that passed the QC were compared against those that were selected using a statistical outlier (SO) removal, a common method used in the UK Biobank imaging studies. That was done to assess to what extent the ML algorithm could generate reliable data without incurring time and resource-consuming VQC.

## Methods

### Study population/data set

UK Biobank was a prospective study of half a million volunteers from the general population aged between 40 and 69, who were recruited between 2006 and 2010^13^ (www.ukbiobank.ac.uk). The UK Biobank imaging study aims to collect brain, heart, and abdomen scans from 100 000 participants, with 70 000 participants scanned so far.^14^ The CMR scanning protocol using a 1.5 T scanner (MAGNETOM Aera, Syngo Platform VD13A, Siemens Healthcare, Erlangen, Germany) has been described in detail elsewhere.^15^ Approximately 50 000 participants were scanned prior to the COVID-19 pandemic.^16^ Around 2000 of these participants, some of whom were infected with COVID-19, and others who were not, were invited back for repeat imaging.^16,17^

Our research group analysed the CMR scans of this COVID-19 UK Biobank sub-study to compare the cardiovascular changes between the baseline imaging visit and repeat imaging visit for those that were infected with COVID-19 and the control group that were not. The characteristics of the 1069 participants whose scans were included in this study are shown in *Table 1*, with their definitions detailed in Supplementary data online, *Table S1*.

**Table 1:** Baseline participants’ characteristics

	Participants (*n* = 1064)
Age, years (mean ± SD)	59.37 ± 7.23
Males (*n*, %)	478 (44.92%)
Ethnicity—Caucasian (*n*, %)	1028 (96.61%)
Ethnicity—other (*n*, %)	36 (3.38%)
Current smoking (*n*, %)	44 (4.12%)
BMI (median + IQR)	26.3 (23.5, 29.3)
Prevalent diabetes (*n*, %)	44 (4.13%)
Prevalent hypertension (*n*, %)	224 (21.05%)

**Figure 1:**
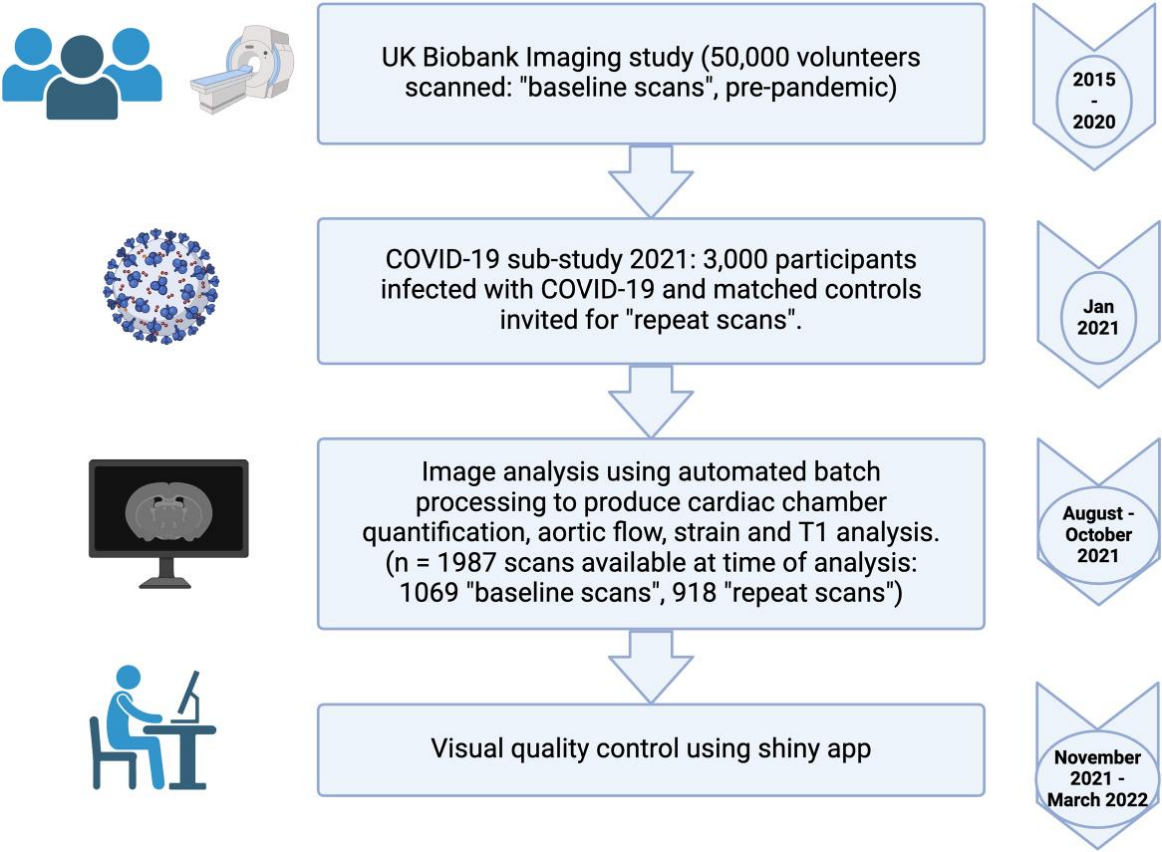
Flow chart detailing timeline of CMR image analysis and quality control process. Created using Biorendr.com.

This paper outlines the automated methods used for large-scale image analysis and the QC process to ensure the accuracy and reliability of these automated methods. *Figure 1* outlines in detail the timeline of image analysis and QC steps.

### CMR image analysis: batch processing

CVI42 software (Circle Cardiovascular Imaging, Inc., prototype 5.14.1.2875) was used to perform an automated batch ML analysis of CMR scans included in the COVID-19 study to derive volumes of cardiac chambers, aortic flow analysis, and native T1. CVI42 (Circle Cardiovascular Imaging, Inc., prototype 5.13.7I) was used to perform an automated batch feature tracking analysis of the same cases to derive myocardial strain. The settings used for each image analysis are detailed below.

Batch processing is a semi-automated process involved preparing ∼300 scans (the maximum the software could execute each time), to be analysed by the automated algorithm imbedded in the new CVI 42 prototypes for the respective type of image analysis. The correct settings had to be selected (detailed below), which was discussed and agreed with experts within the team. The correct destination file paths had to be selected to import each ‘batch’ and for the data output to be safely recorded. All of the automated image analysis generated videos or images of the automated analysis that were used to assess quality.

The resolution of the videos and images was limited to 1024 × 1024 pixels due to storage space, given the large data set. A total of 1987 scans (1069 baseline scans, 918 repeat scans) were analysed and included in the VQC process detailed in this paper.

#### Volumes of cardiac chambers

The whole of the short axis (SAX) stack was chosen to be segmented, where, in the basal slices, only closed contours (i.e. complete circles of both endocardium and epicardium are visible) were included for volume calculation. Simplified endocardial contours without papillary muscle detection [papillary muscles are not identified and therefore not included in the left ventricular (LV) mass calculation] was the protocol for this analysis.

LV endocardial and epicardial contours were placed both in SAX and long axis (LAX) cine images (two-chamber, three-chamber, and four-chamber). Right ventricle (RV) endocardial and epicardial were traced from the SAX and four-chamber cine CMR views using the automated segmentation tool. Left and right atrial (LA and RA) contours were automatically drawn in four-chamber views, and the LA area was also segmented in two-chamber cine CMR view.

#### Aortic flow

The aortic flow was automatically measured using the aortic valve phase contrast image after having defined the inner wall of the aorta in the systolic phase (ROI). A ML algorithm was used to segment the cross-sectional image of the aorta for all images in the sequence independently. Background correction and anti-aliasing background correction was not required due to the high quality of images in UK Biobank.

#### Native T1

CMR native T1 maps were acquired at the mid-ventricular SAX view using the ShMOLLI T1 mapping sequence. A ML algorithm was used to segment the endocardial and epicardial borders into six segments of the native T1 image, which included a 10% offset on both endocardium and epicardium contours. The contoured results are saved as a greyscale image to aid VQC of the algorithm-generated epicardial and endocardial borders, offsets, and myocardial segments.

#### Myocardial strain using feature tracking

The protocol applied temporal smoothing and used LV diastole and systole to guide tracking. LV diastole was set as the reference phase. Any SAX images where there are open LV contours (missing endocardium or epicardium contours) were excluded from the feature tracking. Contours were first drawn with SAX stack segmented only in LV end-diastole and LV end-systole. LAX images were also segmented.

### Visualization of CMR images and videos using a Shiny app

**Figure 2:**
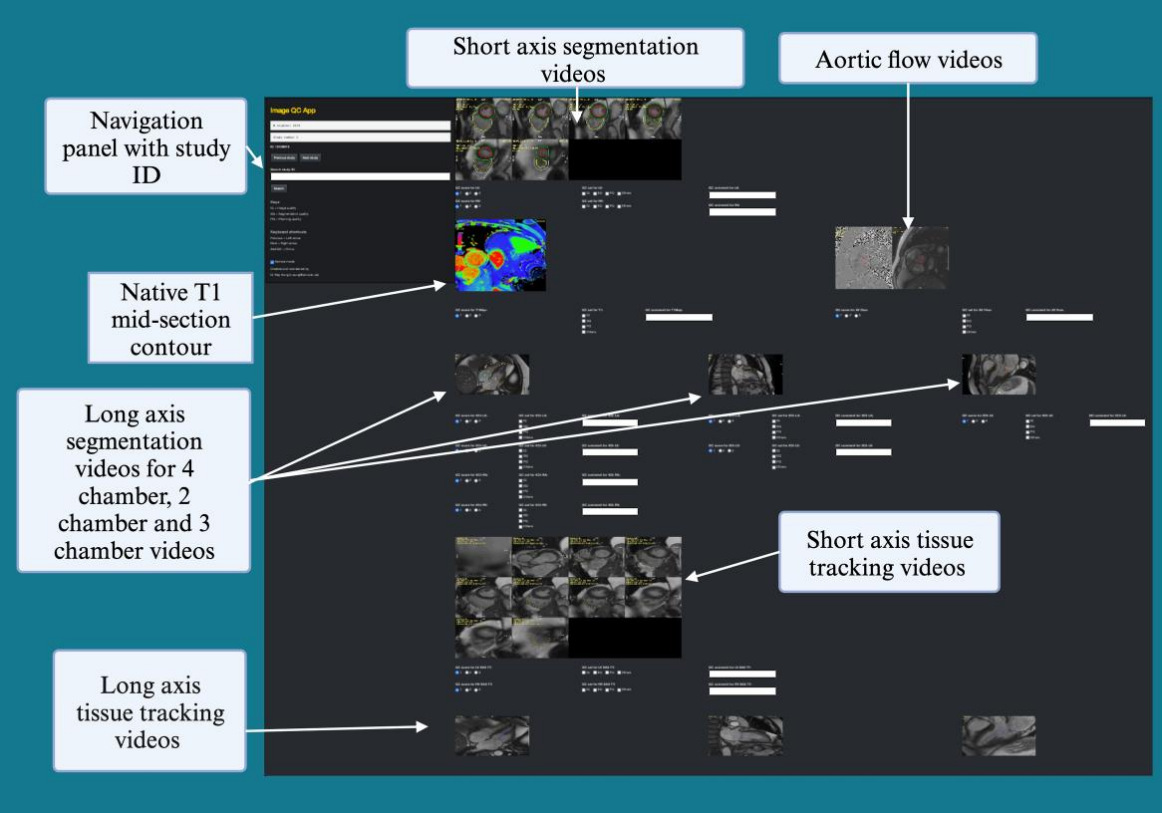
Custom-built shiny app user-friendly interface and layout. Created using Biorendr.com.

We built a custom Shiny^18^ app to perform the visual evaluation of segmentation videos and still images produced by the automated batch processing pipeline. Shiny is an R package designed for the development of interactive web applications and data visualization dashboards. A typical Shiny app consists of a user interface script that controls the layout and appearance of the app and a server script that details the information to build the objects shown in the user interface. The Shiny framework also allows customization by using HTML, CSS, or JavaScript. In this project, we leveraged the ShinyProxy tool^19^ which permits user authentication, wrapped in a Docker^20^ container to deploy our Shiny app for multiple readers. *Figure 2* shows the user-friendly interface of the custom-built Shiny app.

### VQC process

A detailed description of the criteria used to assess the quality and assigning a score of each image analysis modality is provided in *Table 2*.

**Table 2:** Criteria to assess the quality of each image analysis modality

CMR image sequence	QC score 1 (good)	QC score 2 (satisfactory)	QC score 3 (poor)
Contours of cardiac chambers (∼15 videos per scan)			
Points to consider when assessing quality: Accuracy of contouring. Level of operator corrections required to make contours clinically acceptable.			
SAX (RV)	Contours considered of good quality with no operator corrections needed.	Fair or clinically acceptable contours with minor or negligible inaccuracies in the segmentation considered not clinically relevant.	Significantly inaccurate segmentation or requiring significant manual changes to make them clinically acceptable. If an LV or LA severely foreshortened due to planning quality.
SAX (LV)		If an LV or LA somewhat foreshortened due to planning quality.	
4ch (RV)			
4ch (LV)			
2ch (LV)			
3ch (LV)			
4ch (RA)			
4ch (LA)			
Strain (∼ 15 videos per scan)			
Points to consider when assessing quality: Are the contours for end-systole and end-diastole appropriate? Are the tracking markers and the ‘quivers’ (a line which spreads out from the feature dot that is being tracked) appropriately placed in relation to the contours and the ‘quivers’ aligned in the appropriate direction. Look at the ‘extent’ tool—the blue line going from the base to the apex. Is this correct? If the blue line is going outside of the heart, then the data is unlikely to be accurate. If all three long axis images are missing, then this will produce very unreliable strain data and should be classed as grade ‘3’. If >3 points are not tracking appropriately, then the quality of the data will be poor Between 1 and 3, need to look, and if it is related to papillary muscle, then this is inappropriate tracking from the software but could be graded as quality grade 2 if only one tracking marker associated with papillary muscle. The protocol that we have selected should exclude any slices where the blood pool is not visible (apical) or where there are open contours (basal slices) If RA is tracked when tracking the RV then this is a grade 2 and need to enter in free text ‘RA tracked’.			
SAX	Perfect or near perfect	Acceptable (clinically we would include)	Completely inaccurate contours and border points
2ch	Perfect or near perfect	Extent tool (blue line) slightly inaccurate but not outside of the heart	Extent tool (blue line) outside the heart
3ch	Perfect or near perfect	Look at extent tool (blue line) in this image also.	Two or more strain markers tracking papillary muscle & extent tool outside.
4ch	Perfect or near perfect	1–3 markers inappropriate tracking. Slightly inaccurate contours.	Two or more strain markers tracking papillary muscle
Native T1 (1 image)			
Points to consider: Appropriate contours Appropriate colour maps Artefacts in the ROI.			
SAX	Perfect or near perfect	Some irregularities but would be acceptable. If an artefact is in the ROI but can still get an accurate value if manually drawn omitting artefact.	Incorrect contour, colour map/incompatible artefact in ROI.
Aortic valve flow (1 video)			
Aortic valve phase contrast image	Perfect or near perfect contour, no aliasing	Contour slightly inaccurate but acceptable	Incorrect contour and/or aliasing present.

CMR, cardiac magnetic resonance; QC, quality control; SAX, short axis; LV, left ventricle; RV, right ventricle; RA, right atrium; LA, left atrium; 2ch, 2 chamber; 4ch, 4 chamber; ROI, region of interest.

The quality of the automatically derived CMR measurements was assessed by a group of six experienced clinicians in reading CMR (E.R., H.N., J.V., M.Y.K., S.C., and S.E.P.) including three consultant cardiologists with level 3 certification in CMR (M.Y.K., J.V., and S.E.P.) and three well-trained clinical research fellows (E.R., H.N., and S.C.) with >3 years’ experience in analysing CMR. The evaluators were asked to visualize the images and assign quality scores ranging from 1 to 3 (‘good’, ‘satisfactory’, ‘poor’).

Regarding contouring of cardiac chambers, a score of 1 (‘good’) was assigned to good-quality images, with contouring considered of excellent/good quality with no manual changes required. A score of 2 (‘satisfactory’) was given to contouring with minor or negligible inaccuracies that if the scan was being interpreted in a clinical setting would not affect the clinical decisions. A score of 3 (‘poor’) was assigned to either absent contouring, poor-quality images, or gross inaccuracies of the algorithm to segment the cardiac structures significant enough to be considered unacceptable if the scan was interpreted in a clinical setting.

Similar to previous standard operating procedures for contouring analysis, the quality of T1 maps was defined as 1 (‘good’ quality, without motion artefacts), 2 (‘satisfactory’: images are suboptimal but still analysable), and 3 (‘poor’ image quality that makes it not analysable, no image available).

The quality of aortic flow images was judged as 1 (‘good’: images acquired at the correct slice, of good quality, and free of artefacts), 2 (‘satisfactory’: suboptimal images, with minor issues and artefacts but still analysable), and 3 (‘poor’ quality due to incorrect slice location, extreme imaging artefacts, aliasing in the aortic flow, or missing images).

A detailed description of the criteria used to assess the quality and assigning a score of each image analysis modality is provided in *Table 2*.

The QC process comprised three distinct phases. The first QC phase was performed on a sample of 20 CMR cases. Six readers were asked to independently assess the same cases using the quality scoring system previously described. The analysis was followed by a group discussion to assess the inter-observer variability. The discussion also included a review of CMR studies where there was the largest variability in quality scores measured by inter-reader agreement statistics to establish a consensus on the appropriate approach for future such cases.

The second phase of QC included 50 different CMR cases assigned to each observer, followed by another round of group discussion. The observers reviewed challenging cases together, and a consensus was reached to establish a uniform approach to the VQC process.

The third and final phase of the QC was performed on the remaining subset of cases (*n* = 1987) distributed among all the observers, who were asked to score them independently.

### Comparing clinical measures generated from VQC vs. SO removal

We assessed the CMR measures of cardiac structure and function, directly calculated from the automated image segmentations, in a subset of participants (*n* = 1069, using only baseline scans) through two QC methods: VQC and SO removal. Specifically, in this analysis, we assessed the following clinical measures: LV and RV volumes, LV mass, LV ejection fraction (LVEF), LV global longitudinal strain (GLS), aortic flow, and native T1.

The output data from the VQC data set, derived from scans rated as either ‘good’ or ‘satisfactory’, were compared with the data from the SO data set, which were generated by removing outliers three times the interquartile range below the first quartile and above the third quartile. That was done to assess the impact of the QC methods on the clinical measurements. Participants with documented cardiovascular disease were excluded from this analysis. The UK Biobank field IDs used to identify and define those with pre-existing cardiovascular disease can be found in Supplementary data online, *Table S2*.

### Statistical analysis

Continuous variables are presented as proportions and means ± standard deviations. Gwet’s second-order agreement coefficient with ordinal weighting applied (AC2) values was used to assess inter-observer scoring variability.^21^ This method has been validated for use when assessing reliability between multiple operators.^7,21,22^ The coefficient value, 95% confidence interval (CI), and associated *P* values were generated. An agreement coefficient value between 0.61 and 0.80 has been used as an acceptable benchmark in previous literature and by Gwet.^7,23^ Therefore, we have adopted the same limits for this paper. Two-sided *t*-tests were used to determine the significance of the differences in the CMR measurements derived from the VQC and SO data sets for both male and female participants. A *P*  *<* 0.05 was considered statistically significant for all analyses. Statistical analysis was performed using Python Version 3.6.4 (Python Software Foundation, DE, USA).

## Results

### Inter-observer agreement

**Figure 3:**
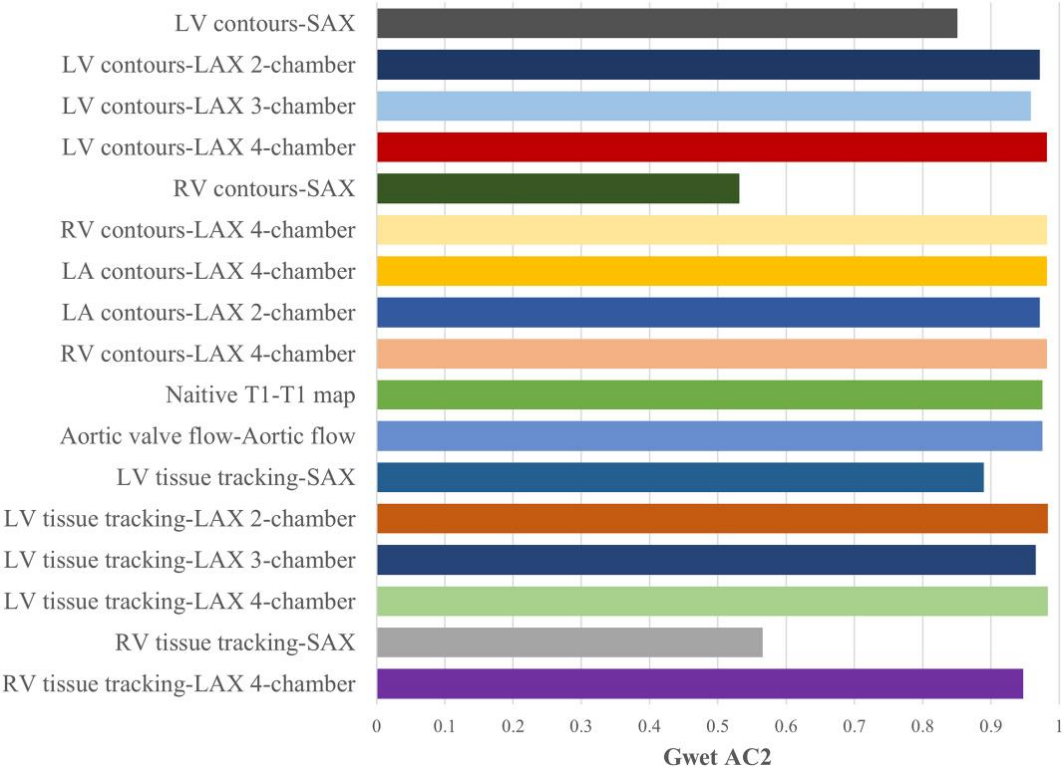
Coefficient (Gwet AC2) by image sequence of scans visually checked by six different operators. The *P* value for all the results shown was <0.05, and the detailed results are shown in Supplementary data online, *Table S3*. Created using Biorendr.com. LV, Left ventricle; SAX, short axis; LAX, long axis; RV, right ventricle; LA, left atrium.

The first phase of the QC process was conducted on a subset of studies (20 scans) equally distributed across the six readers to assess the inter-observer quality score variability using Gwet’s AC2 agreement coefficient with ordinal weighting. The inter-observer agreement for each image sequence is shown in *Figure 3* with the more detailed results seen in Supplementary data online, *Table S3*.

There was substantial inter-observer agreement in quality scoring each image sequence with an overall AC2 score of 0.91 (95% CI 0.84, 0.94), which exceeds the benchmark suggested in the literature.^21^ However, the score agreement was lower for the RV contours in SAX images (AC2 = 0.53, 95% CI 0.42–0.64), and for RV tissue tracking (AC2 = 0.57, 95% CI 0.44–0.69).

### VQC results

#### Contouring of cardiac chambers, native T1 maps, and aortic flow images

Of the 1987 studies selected, we removed those with missing images or clips across each CMR sequence (the highest number of missingness was observed for native T1 maps and aortic flow sequences), and the remaining studies were left to be visually quality-controlled. Of these, the proportions of exams judged of good/acceptable quality (score 1 or 2) were around 99.7%, 99.8%, 98.3%, and 99.8%, for LV, RV, LA, and RA contouring, respectively. Furthermore, the score assigned to each contouring was consistent across all the image sequences (*Table 3*). Similar results were observed for T1 maps and aortic flow analysis, where the proportions of CMR scans scored 1 or 2 were 98.9% and 99.7%, respectively (*Table 3*).

**Table 3:** Distribution of CMR scans and segmentations judged of good or acceptable quality (score 1 or 2) according to the CMR image sequence

CMR image sequence	Proportion of studies with good/acceptable quality
LV contours	
Short axis (SAX) stack	99.8% (*n* = 1976/1980)
Long axis 2-chamber	99.6% (*n* = 1974/1982)
Long axis 3-chamber	99.7% (*n* = 1979/1984)
Long axis 4-chamber	99.8% (*n* = 1981/1985)
RV contours	
Short axis (SAX) stack	99.8% (*n* = 1977/1980)
Long axis 4-chamber	99.9% (*n* = 1983/1985)
LA contours	
Long axis 4-chamber	99.6% (*n* = 1978/1985)
Long axis 2-chamber	97.0% (*n* = 1965/1982)
RA contours	
Long axis 4-chamber	99.8% (*n* = 1981/1985)
Native T1 maps	98.9% (*n* = 1909/1930)
Aortic valve flow	99.7% (*n* = 1923/1928)

The proportion of studies deemed of good/acceptable quality was calculated per each image sequence after removing the studies with missing images or clips, which were due to that image sequence not being performed for those studies or corrupted files not being recognized by software.

LV, left ventricle; RV, right ventricle; LA, left atrium; RA, right atrium.

The CMR image sequence judged as of poor quality (score 3) had either poor imaging quality (e.g. artefacts, aliasing for aortic flow sequence) or plane quality issues (e.g. images foreshortened) or incomplete/failed segmentation (see Supplementary data online, *Table S4*).

#### Strain analysis

Scans from the COVID-19 cohort (*n* = 1987) were visually quality-checked, 1069 of these scans were baseline scans and 918 were repeat scans. The results can be seen in *Table 4*. The scores given during the visual quality check for the automated image analysis to produce strain data were examined to determine the overall quality of the automated batch image analysis using this software.

**Table 4:** The percentage of 1987 scans and segmentations visually scored to be good or satisfactory (grade 1 or 2, respectively) for each image sequence used for strain analysis

CMR image sequence	Proportion of studies with good/acceptable quality
LV tissue tracking	
Short axis (SAX) stack	90.2% (*n* = 1788/1982)
Long axis 2-chamber	97.3% (*n* = 1912/1966)
Long axis 3-chamber	97.6% (*n* = 1917/1964)
Long axis 4-chamber	97.4% (*n* = 1921/1973)
RV tissue tracking	
Short axis (SAX) stack	99.5% (*n* = 1972/1982)
Long axis 4-chamber	98.4% (*n* = 1956/1987)

The proportion of studies deemed of good/acceptable quality was calculated per each image sequence after removing the studies with missing images or clips, which were due to that image sequence not being performed for those studies or corrupted files not recognized by the software.

LV, left ventricle; RV, right ventricle.

LAX image feature tracking and RV SAX were >96% of the time graded as being either excellent or good (grade 1 or 2, respectively). The LV-SAX tissue tracking score was comparatively lower at 90% for being either good or satisfactory.

We examined the score and comments for the 1982 scans where LV SAX tissue tracking was available to further understand why the LV-SAX tissue tracking image sequence had comparatively a lower proportion graded as good or satisfactory or poor. Given the score of ‘good’ or ‘satisfactory’ (1 or 2) were 90.2% (1788 scans) and 9.8% (194 scans) were given the score of poor.^3^ The QC comments revealed that there was only partial tracking of LV in some slices in 24 of these scans. The comments also revealed that 170 scans were graded as poor quality^3^ due to missing any feature tracking markers on the LV in SAX stack images. Lack of feature tracking on the LV in SAX stack images would result in lack of data output for global circumferential strain (GCS) and global radial strain (GRS) as these are derived from LV myocardial tracking in the SAX images. Therefore, the grade of poor quality^3^ in this case would be redundant since there is no clinical output data derived from these images.

We examined the data output that revealed that 64 of the scans graded as poor quality and missing LV tracking in SAX stack did not have any output for GCS and GRS, as predicted, making the score of poor quality redundant. Therefore, the total number of scans that is valid to be included in the assessment is 1918 (1982–64). The remaining 106 scans (170–64), which were given a ‘poor’ score, were visually re-checked and showed that LV tracking was present in all these scans, but were difficult to visualize at a glance (reduced quality image output which was necessary due to limited storage capacity for larger data set). Therefore, these scans correctly had corresponding data output for GCS and GRS. Only 6 of these 106 scans had truly ‘poor’ LV tracking limited to few LV slices deserving the score of 3 when visually re-checked.

Therefore, there were only 30 scans (24 with only partial LV tracking and 6 from the repeat review round of visual QC) where the score of ‘poor’ quality^3^ was appropriate, with the other 64 scan scores no longer applicable to be included in the total, as there was appropriately no corresponding data output when the LV is not tracked in the short axis images. This leads us to reclassify the scores and 98.4% (1888/1918) of the feature tracking for LV SAX tissue tracking images are of either good or satisfactory quality. The score was incorrectly reduced due to human discrepancy in the visual quality checking process.

### Comparing clinical measures: SO removal vs. VQC data sets

When comparing the CMR measures between the VQC data set and the SO data set, we found no significant differences in any of the clinical measures related to cardiac structure and function, including LV and RV volumes, LV mass, LVEF, LV GLS, aortic flow, and native T1 (*Table 5*). Additionally, our results showed no significant differences between the two data sets when analysing the measures separately by sex. This suggests that the use of either VQC or SO methods in selecting data for analysis did not have a significant impact on the results obtained.

**Table 5:** CMR measures at baseline imaging visit of healthy men and women in the COVID-19 imaging study

CMR measure	Male (SO data set) (mean ± sd)	Male (VQC data set) (mean ± sd)	*P* value	Female (SO data set) (mean ± sd)	Female (VQC data set) (mean ± sd)	*P* value
LV end-diastolic volume (mL)	167.7 ± 30.1	167.5 ± 29.8	0.91	130.6 ± 22.3	130.9 ± 22.6	0.83
LV end-systolic volume (mL)	68.9 ± 16.2	69.0 ± 16.5	0.91	49.5 ± 11.4	49.6 ± 11.5	0.86
LV mass (g)	107.9 ± 17.8	108.0 ± 18.0	0.93	74.2 ± 12.7	74.5 ± 12.8	0.77
LV ejection fraction (%)	59.1 ± 5.4	58.9 ± 5.9	0.62	62.2 ± 5.2	62.2 ± 5.3	0.9
LV global longitudinal strain (%)	−17.5 ± 2.0	−17.4 ± 2.3	0.62	−18.8 ± 2.0	−18.8 ± 2.1	0.67
RV end-diastolic volume (mL)	177.9 ± 32.0	177.3 ± 31.7	0.78	132.5 ± 25.0	132.7 ± 25.1	0.92
RV systolic volume	104.1 ± 21.0	103.8 ± 20.8	0.85	82.9 ± 15.8	83.0 ± 15.8	0.93
Aortic total forward volume (mL)	59.7 ± 38.4	59.5 ± 38.2	0.28	52.1 ± 30.7	52.5 ± 30.6	0.84
Native T1	912.4 ± 33.4	920.2 ± 143.3	0.95	943.1 ± 41.2	942.3 ± 47.1	0.76

CMR measures from one data set were generated after removing statistical outliers (SO data set) and the other data set for the same participants were generated from scans that were scored ‘good’ or ‘satisfactory’ as part of the VQC data set process. The sample number included varies according to CMR measure due to varied missingness of CMR image sequences from which they were generated. In the statistical outlier data set, the number of men included varied between 409 and 450, and number of women included varied between 518 and 574. In the visually quality-checked data set, the number of men varied between 439 and 478, and the number of women varied between 532 and 585. *P* values based on paired *t*-tests.

CMR, cardiac magnetic resonance; SO, statistical outlier; VQC, visual quality control; LV, left ventricle; RV, right ventricle.

## Discussion

This paper is the first, to our knowledge, that outlines the QC of automated batch processing performed on a large scale for research purposes. Across all images reviewed, there were <5% that were graded ‘poor’ quality. The results demonstrate the reliability of automated image analysis using the batch processing prototypes developed by Circle Cardiovascular Imaging, Inc. (CVI42 prototypes). Other studies have also demonstrated the superior accuracy and reproducibility of automated image analysis methods using ML algorithms.^4,11,24^ Our findings illustrate the impact of human error that can lead to the scan quality being inappropriately downgraded especially with the tissue tracking analysis for LV-SAX where tracking markers were difficult to visualize.

During the first phase of the QC process the results show there was, overall, substantial inter-operator agreement, except for the contouring of RV SAX images. This reflects the reality of CMR image analysis where there is still much debate in both research and clinical settings about the best way to contour/track the right ventricle.^25^ These images were the focus of the subsequent group huddle session to establish a consensus between all the operators for the next and final phases of QC. There was agreement with regard to RV tracking, and this did not require extensive discussion to reach a consensus.

The comparison of CMR measurements generated from the VQC data set and the SO data set is also encouraging. There were no statistically significant results when investigating whether the clinical data output differed between the two data sets. Therefore, our findings suggest a statistical approach to remove outliers of image-derived phenotypes is sufficient in UK Biobank image analysis and may save time of experts performing visual quality checks on segmentations.

The QC processes can be time- consuming and labour- intensive. However, it is currently necessary to safeguard the quality of the data output until the reliability of these automated methods is established and widely accepted. The use of online platforms such as the one developed in-house for this study can greatly improve the efficiency and ease of the QC process.^26^ The Shiny app allowed multiple operators to contribute to the process without being bound to a physical space or restricted timings. Therefore, the QC of ∼2000 scans which meant visually reviewing ∼62 000 videos and ∼2000 images, was completed efficiently (median time spent per scan overall was 32 s ranging between operators from 20–56 s), whilst also being convenient for all the operators. The use of the Shiny app also facilitated intense huddle discussions that could be reviewed remotely at the beginning to ensure consistency in scoring. Overall, the time and expertise spent on this QC process is relatively small compared to the time it would have taken for the large number of scans included in this study to be manually analysed and may still need to go through a quality check process. Validating the use of automated image analysis methods is an essential step to allow progress to be made using large data sets that include imaging (for example the UK Biobank imaging data set with ∼50 000 scans to date), which could lead to interesting and previously unknown knowledge in research.

Automated image analysis will also allow greater efficiency in clinical settings where automated analysis can be relied upon for the more mundane task of producing the quantitative data and the expert clinician can then spend more time on the interpretation. This has already been reported in other studies.^4,24^ The findings of this paper would suggest that even for more complex image analysis such as aortic flow, native T1, and feature tracking strain, we can rely on automated processes to produce reliable data. The clinicians will always need to be aware of the quality checking parameters such as those described in *Table 2* for each imaging modality when it comes to interpretation, particularly if there are discrepancies in the expected data output and the actual data output. However, if the prototypes described in this paper are used for clinical or research purposes, there can be overall high confidence in the results that are produced based on the results described above.

### Strengths and limitations

This study outlines a robust visual QC process, which can be easily replicated by others, using an online platform which makes the process efficient and convenient even with multiple operators. The results are promising and promote the use of automated image analysis methods as reliable and accurate with the overwhelming majority (>95%) of the analysis deemed to be of ‘good’ or ‘satisfactory’ quality. This allows us to recommend that we continue to use automated batch processing image analysis methods on a larger scale without the need for constant, time- and resource-consuming visual quality checks, and instead, the removal of statistical outliers is sufficient. The comparison made between CMR measures generated after the two types of QC demonstrates this recommendation in practice, where there is no difference in the overall data output from both QC methods. Future work could also compare visual quality checks with automated QC algorithms described in the literature.^9,27^ This would also be an efficient method for quality-checking the automated image analysis.

However, one limitation is that the encouraging results seen in this paper are limited to scans performed in the UK Biobank imaging study where standardized protocols are implemented, which ensure a relatively high standard of images to begin with. In addition, the comparison made between the visually quality-checked data set and SO data set is limited to those without cardiovascular disease only, after those with overt cardiovascular pathology were excluded. Therefore, variation in data output may exist when those with conditions such as severe heart failure and cardiomyopathies are included, which would lead to more extreme data values being generated. These may then be erroneously removed using the SO method, whereas visual quality checks would note that the extreme values are a reflection of the disease rather than a true erroneous result. This is a key limitation of this secondary analysis in this study.

Finally, the automated batch processing software was provided by Circle and is, therefore, not an open-source resource available to anyone. However, the findings are still encouraging regarding the potential for automated image analysis to be a viable time-saving tool with reliable output.

## Conclusion and recommendations

This detailed and comprehensive QC process of the COVID-19 study scans has shown that >95% of the UK Biobank scans analysed using CVI42 batch processing methods are of good or satisfactory quality. Therefore, for the larger imaging cohort of up to ∼100 000 studies at baseline and 60 000 at a follow-up, which will be analysed using the same CVI42 batch processing tools, extensive visual quality checks are not required. The recommendation would be that removal of extreme statistical outliers and non-sensical data is sufficient to ensure a good quality for the data outputs. However, when the research question involves looking at a more specific disease affected cohort, additional visual quality check of studies identified as being statistical outliers may be beneficial so that data reflective of true pathology is not inadvertently removed.

Further validation of automated batch processing image analysis will need to be performed for other cohorts and clinical scans before these recommendations can be applied on a wider basis. The robust visual quality check process and the online platform (Shiny app) described in this paper could be beneficial for future work in this field.

## Supplementary Material

qyae094_Supplementary_Data

## Data Availability

This research was conducted using the UKB resource under access application 2964. UK Biobank will make the data available to all researchers for all types of health-related research that is in the public interest, without preferential or exclusive access for any persons. All researchers are subject to the same application process and approval criteria as specified by UK Biobank. For more details on the access procedure, see the UK Biobank website: http://www.ukbiobank.ac.uk/register-apply/.

## Lead author biography

Steffen Petersen is a Professor of Cardiovascular Medicine at Queen Mary University of London and a Consultant Cardiologist at Barts Health NHS Trust. He is the British Heart Foundation Data Science Centre Deputy Director and President of the European Society of Cardiology's European Association of Cardiovascular Imaging. Petersen holds medical degrees from Germany and the UK, as well as a Master’s in Public Health from Harvard. He is level 3 certified in cardiovascular magnetic resonance and has over 20 years of experience in this field. His research focuses on cardiac imaging, population studies, and cost-effectiveness analysis in prevention.

Dr Nay Aung is a Consultant Cardiologist at Barts Heart Centre, St Bartholomew’s Hospital in London, and a Senior Clinical Lecture and MRC Clinician Scientist at Queen Mary University London. He has clinical sub-specialty interest in heart failure and cardiac imaging. His research focuses on deep phenotyping of cardiac images using artificial intelligence techniques and the application genomics to understand the determinants of adverse cardiac remodelling. He is an invited speaker for international conferences and serves on multiple committees in the European Society of Cardiology (ESC).
