# Supplementary material for: Quality control of cardiac magnetic resonance imaging segmentation, feature tracking, aortic flow, and native T1 analysis using automated batch processing in the UK Biobank study
Source: Eur Heart J Imaging Methods Pract. 2024 Sep 16;2(3):qyae094. doi: 10.1093/ehjimp/qyae094 (PMC11462446; doi:10.1093/ehjimp/qyae094)
Supplement: qyae094_Supplementary_Data [file qyae094_supplementary_data.docx]

**SUPPLEMENTARY MATERIAL**

**Supplementary Table 1: UK Biobank field IDs used for Baseline Participants characteristics**

|  | UK Biobank field description | Source UKBB field ID (codes used) |
| --- | --- | --- |
| Age, years (mean ±SD) | Age at assessment centre attendance | 21003 |
| Males (n, %) | - | 31 |
| Ethnicity – Caucasian (n, %) | - | 21000 |
| Ethnicity – other (n, %) | - | 21000 |
| Current smoking (n, %) | - | 20116 (current, value=2) |
| BMI (median + IQR) | - | 23104 |
| Prevalent diabetes (n, %) | Self-reported | 20002 ('1220', '1222', '1223') |
|  | Medication | Medication: 6177 (value=3) |
|  | ICD 10 | 41202 ('E100', 'E101', 'E102', 'E103', 'E104', 'E105', 'E106', 'E107', 'E108', 'E109', 'E110', 'E111', 'E112', 'E113', 'E114', 'E115', 'E116', 'E117', 'E118', 'E119', 'E130', 'E131', 'E132', 'E133', 'E134', 'E135', 'E136', 'E137', 'E138', 'E139', 'E140', 'E141', 'E142', 'E143', 'E144', 'E145', 'E146', 'E147', 'E148', 'E149', 'G590', 'G632', 'H280', 'H360', 'M142', 'N083', 'O240', 'O241', 'O243', 'O244', 'O249', 'Y423'] |
| Prevalent hypertension (n, %) | Self-reported | 20002 ('1065', '1072') |
|  | Medication | Medication: 6177 (value=2) |
|  | ICD10 | 41202 ('I10X', 'I110', 'I119', 'I120', 'I129', 'I130', 'I131', 'I132', 'I139') |

**Supplementary Table 2: UK Biobank field IDs used to identify those with cardiovascular disease**

| **Source** | | **UK Biobank field** | | **Description** |
| --- | --- | --- | --- | --- |
| ***Heart Failure & Cardiomyopathies*** | |  | |  |
| ICD10 Summary diagnoses | | 41270, 41280 | | I50 Heart failure  I42 Cardiomyopathy  I43 Cardiomyopathy in diseases classified elsewhere |
|  | |  | | I11.0 Hypertensive heart disease with (congestive) heart failure  I13.0 Hypertensive heart and renal disease with (congestive) heart failure  I13.2 Hypertensive heart and renal disease with both (congestive) heart failure and renal failure  I25.5 Ischaemic cardiomyopathy |
| First occurrences | | 131354 | | Date first diagnosis: Heart failure |
|  | | 131338 | | Date first diagnosis: Cardiomyopathy |
|  | | 131340 | | Date first diagnosis: Cardiomyopathy in diseases classified elsewhere |
|  | | 131288 | | Date first diagnosis: Hypertensive heart disease |
|  | | 131292 | | Date first diagnosis: Hypertensive heart and renal disease |
|  | | 131306 | | Date first diagnosis: Ischaemic cardiomyopathy |
| ***Myocardial infarction*** |  | |  | |
| ICD10 Summary diagnoses | | 41270, 41280 | | I21 Acute myocardial infarction |
|  | |  | | I22 Subsequent myocardial infarction |
|  | |  | | I23 Certain current complications following acute myocardial infarction |
|  | |  | | I24.1 Dressler's syndrome |
|  | |  | | I25.2 Old myocardial infarction |
| First occurrences | | 131298 | | Date first diagnosis: Acute myocardial infarction |
|  | | 131300 | | Date first diagnosis: Subsequent myocardial infarction |
|  | | 131302 | | Date first diagnosis: Certain current complications following acute myocardial infarction |
|  | | 131304 | | Date first diagnosis: Other acute ischaemic heart diseases |
|  | | 131306 | | Date first diagnosis: Chronic ischaemic heart disease |
| Diagnosed by doctor | | 3894 | | Age heart attack diagnosed |
| Algorithmically defined | | 42000 | | Date of myocardial infarction |

**Supplementary Table 3: Gwet’s AC2 scores, associated 95% confidence intervals and p values for each image sequence of 20 scans and segmentations visually quality checked by six different operators.**

| **CMR measure** | **Gwet Agreement Coefficient value [95% Confidence interval]** | **P-value** |
| --- | --- | --- |
| **LV contours** |  |  |
| Short axis stack (SAX) | 0.85 [0.75 - 0.95] | <0.001 |
| Long axis 2-chamber | 0.97 [0.91 - 1] | <0.001 |
| Long axis 3-chamber | 0.96 [0.89 - 1] | <0.001 |
| Long axis 4-chamber | 0.98 [0.94 - 1] | <0.001 |
| **RV contours** |  |  |
| Short axis stack (SAX) | 0.53 [0.42 - 0.64] | <0.001 |
| Long axis 4-chamber | 0.98 [0.94 - 1] | <0.001 |
| **LA contours** |  |  |
| Long axis 4-chamber | 0.98 [0.94 - 1] | <0.001 |
| Long axis 2-chamber | 0.97 [0.91 - 1] | <0.001 |
| **RA contours** |  |  |
| Long axis 4-chamber | 0.98 [0.94 - 1] | <0.001 |
| **Native T1** |  |  |
| T1 map score | 0.98 [0.93 - 1] | <0.001 |
| **Aortic valve flow** |  |  |
| Aortic flow score | 0.98 [0.93 - 1] | <0.001 |
| **LV tissue tracking** |  |  |
| Short axis stack (SAX) | 0.89 [0.79 - 0.99] | <0.001 |
| Long axis 2-chamber | 0.99 [0.95 - 1] | <0.001 |
| Long axis 3-chamber | 0.97 [0.91 - 1] | <0.001 |
| Long axis 4-chamber | 0.98 [0.95 - 1] | <0.001 |
| **RV tissue tracking** |  |  |
| Short axis stack (SAX) | 0.57 [0.44 - 0.69] | <0.001 |
| Long axis 4-chamber | 0.95 [0.89 - 1] | <0.001 |

**Supplemental Table 4. Reasons of assigning a score of 3 based on CMR image sequence**

| **CMR image sequence** | **Comments for scoring 3** |
| --- | --- |
| **LV contours** |  |
| Short axis stack (SAX) | Absent/failed apical segmentation, incomplete SAX stack |
| Long axis 2-chamber | Image quality |
| Long axis 3-chamber | Foreshortened LV |
| Long axis 4-chamber | Foreshortened LV |
| **RV contours** |  |
| Short axis stack (SAX) | Incomplete SAX stack |
| Long axis 4-chamber | Foreshortened RV |
| **LA contours** |  |
| Long axis 4-chamber | Foreshortened LA |
| Long axis 2-chamber | Foreshortened LA |
| **RA contours** |  |
| Long axis 4-chamber | Foreshortened RA |
| **Native T1 maps** | Artefacts |
| **Aortic flow** | Aliasing |
